# Spirituality as a Positive Youth Development Construct: A Conceptual Review

**DOI:** 10.1100/2012/458953

**Published:** 2012-04-24

**Authors:** Daniel T. L. Shek

**Affiliations:** ^1^Department of Applied Social Sciences, The Hong Kong Polytechnic University, Hong Kong; ^2^Public Policy Research Institute, The Hong Kong Polytechnic University, Hong Kong; ^3^Department of Social Work, East China Normal University, Shanghai 200241, China; ^4^Kiang Wu Nursing College of Macau, Macau, China; ^5^Division of Adolescent Medicine, Department of Pediatrics, Kentucky Children's Hospital, University of Kentucky College of Medicine, Lexington, KY 40506-9983, USA

## Abstract

The concept of spirituality as a positive youth development construct is reviewed in this paper. Both broad and narrow definitions of spirituality are examined and a working definition of spirituality is proposed. Regarding theories of spirituality, different models pertinent to spiritual development and the relationship between spirituality and positive youth development are highlighted. Different ecological factors, particularly family and peer influences, were found to influence spirituality. Research on the influence of spirituality on adolescent developmental outcomes is examined. Finally, ways to promote adolescent spirituality are discussed.

## 1. Background

There are research studies showing that spiritual and religious involvement is an important dimension in adolescent development. For example, Gallup and Bezilla reported that 95% of American adolescents believed in God [1]. Based on the data collected from “The Project Teen Canada”, Bibby found that 75% of the respondents regarded themselves as members of a religion, 60% viewed spirituality as important, and 48% indicated that they had spiritual needs [2]. In a study based on 112,232 freshmen in 236 colleges and universities in the United States, Astin et al. reported that 77% of the students agreed that they were “spiritual beings” and roughly four-fifth of them indicated that they had interest in spirituality and they believed in the sacredness [3]. These findings are consistent with the view of Benson and Roehlkepartain [4] that “most young people view spiritual development as an important part of their lives” (p. 14). King and Boyatzis [5] similarly commented that adolescence “may be a particularly important time period in which to study spiritual and religious development” (p. 2).

Using life meaning as an illustration, adolescents tend to think in abstract terms and explore future possibilities when they are cognitively mature. They commonly ask questions about life, including the following: What is the meaning of life? What is a meaningful life? Why do we exist? What should we accomplish in life? These questions commonly fall within the large scope of “meaning of life” or “purpose in life”, which addresses three interrelated issues which are the meaning of life (e.g., what life signifies, personal reasons, and importance of existence), meaningfulness of life, (e.g., whether life is worth living or purposeful), and purpose in life (e.g., life goals, life purpose, things to be accomplished, ideals to be attained). The importance of the meaning of life in adolescent behavior is clearly reflected in human history. For example, in the 1930s, young people supported Hitler in Nazi Germany when they believed that building an ethnically superior Germany was their life mission. During the Cultural Revolution in Communist China in the 1960s, the Red Guards fiercely fought against “enemies” of the proletarians when they saw that building a Communist utopia was their sacred life goal. In the contemporary world, many young people in Africa participate in military activities to look for changes for their countries.

Unfortunately, despite the importance of spirituality in adolescent development, a review of the literature showed that less than 1% of the literature on children and adolescents had examined issues on spirituality and religiosity [6]. As commented by King and Boyatzis [5], “adolescents' spirituality and religion have been relatively neglected in the developmental sciences” (p. 2). In addition, there is a huge research gap in the study of spirituality in the clinical literature [7]. Against this background, this paper attempts to review the concept of spirituality in adolescence. Besides definitions and theories, antecedents of adolescent spirituality and its effects on developmental outcomes are presented. Finally, ways to promote adolescent spirituality are presented.

## 2. Definition of Spirituality

Various definitions of spirituality have been put forward by different researchers. Based on qualitative analyses of various definitions of religiousness and conceptions of spirituality, Scott reported that the conceptions were distributed over nine content areas, with no category containing most of the definitions [8, 9]. These content areas include (1) connectedness or relationship, (2) processes contributing to a higher level of connectedness, (3) reactions to sacred or secular things, (4) beliefs or thoughts, (5) traditional institutional structures, (6) pleasurable existence, (7) beliefs in the sacred or higher being, (8) personal transcendence, and (9) existential issues and concerns. Markow and Klenke pointed out that there were more than 70 definitions of spirituality at work [10].

Perhaps the first clarification that should be made is the distinction between spirituality and religion. Pargament [11] argued that religion is “the search for significance in ways related to the sacred” whereas spirituality is “the search for the sacred” (pp. 11-12). Worthington et al. [12] defined religion as “adherence to a belief system and practices associated with a tradition in which there is agreement about what is believed and practiced” whereas spirituality as “a more general feeling of closeness and connectedness to the sacred. What one views as sacred is often a socially influenced perception of either (a) a divine being or object or (b) sense of ultimate reality or truth” (p. 205). With reference to this conception, while religion is related to institutional beliefs and the sacred, the divine and institutional religion is not necessarily related to the definition of spirituality. According to Worthington et al., there are four types of spirituality, with the first one more related to religion: religious spirituality (closeness and connection to the sacred defined by religion), humanistic spirituality (closeness and connection to mankind), nature spirituality (closeness and connection to nature), and cosmos spirituality (closeness and connection to the whole of creation) [12]. In the project on the role of spirituality in higher education at the Higher Education Research Institute at the UCLA, Austin and his associates [3] distinguished spiritual attributes and religious attributes. While spiritual attributes include spiritual quest (answers to life's questions), ecumenical worldview (transcendence of ethnocentrism and egocentrism), ethics of care (compassion), charitable involvement (services to others), and equanimity (inner peace), religious attributes include religious commitment (degree of identification with the religion), engagement (behavioral aspect of religion), conservatism (identification with orthodox beliefs), skepticism (questions raised on the beliefs), and struggle (unsettlement about religion).

Broad as well as narrow definitions of spirituality exist in the literature. An example of a broad definition was put forward by Myers et al., [13] who defined spirituality as “personal and private beliefs that transcend the material aspects of life and give a deep sense of wholeness, connectedness, and openness to the infinite” (p. 265). According to this conception, spirituality includes (a) belief in a power beyond oneself, (b) behavior in relation to the infinite such as prayer, (c) meaning and purpose of life, (d) hope and optimism, (e) love and compassion, (f) moral and ethical guidelines (g) transcendental experience. Another broad definition can be seen in Lewis who conceived spirituality as the life affirmed in a relationship with God, self, community, and environment which leads to the nurturance and celebration of wholeness [14]. Within this context, spiritual needs include meaning, purpose and hope, transcendence circumstances, integrity and worthiness, religious participation, loving and serving others, cultivating thankfulness, forgiving and being forgiven, and preparation for death and dying. On the other hand, there are relatively narrower definitions of spirituality such as focus on existential or transcendental questions, belongingness to involvement of cardinal values underlying every aspect of life, and self-reflective behavior. For example, Worthington et al. [12] conceived spirituality as “general feeling of closeness and connectedness to the sacred” (p. 205).

An integration of the literature shows that several elements are commonly employed in the definition of spirituality. These include meaning and purpose of life, meaning of and reactions to limits of life such as death and dying, search for the sacred or infinite, including religiosity, hope and hopelessness, forgiveness, and restoration of health [15]. Lau pointed out that three key elements of spirituality had been identified in the literature [16]. The first element is horizontal as well as vertical relationships in human existence [17]. While horizontal relationships are related to oneself, others, and nature, vertical relationship involves a transcendental relationship with a higher being. The second element is beliefs and values which are integral to answers to spiritual questions such as life and death. The third element is the meaning of life. In this paper, a broader conception of spirituality (i.e., horizontal and vertical relationships, beliefs, meaning of life) is adopted.

## 3. Assessment of Spirituality

Two broad strategies are commonly used to assess the construct of spirituality: quantitative approach and qualitative approach. To maximize the strengths and minimize the limitations of both approaches, researchers commonly use both approaches to assess spirituality. In the quantitative approach, either single items or scales are used to assess spirituality. For example, researchers have used single items to assess a respondent's ranking of the importance of things in life, such as wealth, family, health, friends, social status, and peace of mind. Also commonly, researchers use a few items to assess religiosity and religious involvement. Obviously, both single-item measure and multiple-item measures are problematic because their reliability and validity are usually not examined. To overcome such problems, psychological scales have been developed to measure the construct of spirituality. Some examples include the Spiritual WellBeing Scale, Purpose in Life Questionnaire, Templer's Death Anxiety Scale, Enright Forgiveness Inventory, and Herth Hope Index. Unfortunately, there are few validated measures of spirituality for Chinese adolescents [15, 18]. Furthermore, few researchers use advanced statistical techniques such as structural equation modeling to assess spirituality.

Qualitative methods (such as open-ended questions, drawing, verbal commentary techniques, and case studies) are also employed to examine spirituality, particularly in the clinical settings. The common features of qualitative research include naturalistic inquiry, inductive analysis, holistic perspective, qualitative data, personal contact and insight, dynamic system, unique case orientation, empathetic neutrality, and design flexibility. For example, children have been invited to draw pictures about their attitudes towards death and dying. While qualitative study can capture the perspectives of the informants and is a more naturalistic form of research, it is often criticized as biased and polluted by ideological preoccupations. As such, ways to enhance the credibility of data collection, analyses, and interpretations are important issues to be considered.

## 4. Theories of Spirituality

There are three categories of theories of spirituality. The first category of theories focuses on the nature of spirituality in relation to different aspects of human development. For example, there are theories suggesting that spirituality is part of quality of life. In the model of psychological well-being proposed by Ryff and Singer [19], meaning, purpose, growth, and self-actualization are basic components of well-being, and psychological well-being includes self-acceptance, environmental mastery, positive relations with others, purpose in life, personal growth, and autonomy. In the Wellness Model proposed by Adams et al., emotional centeredness, intellectual stimulation, physical resilience, psychological optimism, social connectedness, and spiritual life purpose are basic dimensions [20].

The second group of theories concerns the nature of spiritual development. In Erikson's theory of psychosocial development [21], the major task of an adolescent is to develop an identity, with ego identity versus role confusion as the basic psychosocial crisis. In Marcia's framework, crisis and commitment are two basic dimensions of identity, particularly in religious or spiritual identity [22]. In the spiritual development model proposed by Fowler [23], there are six stages of faith development, with Stage 3 and Stage 4 most relevant to spiritual development of adolescents. In Stage 3, faith development takes the form of “synthetic-conventional” faith which is characterized by conformity with little reflection on one's religious beliefs. This stage is quite typical in the Chinese culture. In Stage 4, “individuative-reflective” faith is characterized by personal struggle and choice. It is argued that the existence of personal struggle and choice are important elements of mature spirituality.

In the faith development model suggested by Genia [24], five stages were proposed. Following the stages of Egocentric Faith (Stage 1) and Dogmatic Faith (Stage 2), the third stage is Transitional Faith where adolescents can critically examine their spirituality which is prompted by adolescents' gradual maturation in cognitive ability and interpersonal perspective taking. If the transition in Stage 3 is successful, adolescents will progress to Stage 4 (Reconstructed Internalized Faith) and Stage 5 (Transcendent Faith) where transcendent faith is characterized by flexible system of faith, universal principles, and permeable psychospiritual boundaries.

The third group of theories is on the relationship between spirituality and positive youth development. In the model proposed by Benson [25], there are 40 developmental assets in adolescent development, where life meaning and positive beliefs are important internal assets that influence adolescent development. Dowling et al. proposed a model in which spirituality was hypothesized to influence thriving with religiosity as a mediating factor [26]. In a review of 77 positive youth development programs in the United States, Catalano et al. concluded that positive youth development constructs are intrinsic to the successful programs, with spirituality as one of the constructs identified which is defined as the development of purpose and meaning in life, hope, or beliefs in a higher power [27]. There are many recent publications highlighting the relationship between positive youth development and spirituality [28–30].

## 5. Antecedents of Adolescent Spirituality

Benson and Roehlkepartain [4] concluded three processes intrinsic to adolescent spirituality. The first process is awareness or awakening which contributes to the development of spiritual identity, meaning, and purpose. The second process is interconnecting or belonging which involves seeking or experiencing relationships with others, including divine beings. The final process is a way of life where a person expresses one's spiritual identity through different activities and relationships. This model further proposed that these three processes shaping adolescent developmental outcomes are related to other dimensions of development which are influenced by context (e.g., family, peers, and neighborhood), culture (e.g., media), and metanarratives (e.g., stories). Besides ecological models, there are other accounts on the factors influencing adolescent spirituality. For the channeling hypothesis, it is stated that children are “channeled” into different social groups based on the religious expectations of the parents [31]. The spiritual modeling perspective based on the social learning premise indicates that adolescents model their religious behavior of their parents [32]. The role of significant others in shaping adolescent spirituality is also highlighted by Fry who explicitly stated that “it is through supportive and sharing relationships within a trusting and accepting atmosphere that the adolescent gains the courage to explore what experiences make sense or providing meaning even in the face of doubts” (p. 98), thus emphasizing the role of intimate relations in the development of adolescent purpose in life [33].

There are research findings showing that family and peers exert influence on the spiritual development in adolescents. In a longitudinal study based on individuals, parents, peers, schools, and community, Regnerus et al. found that while parents and friends strongly influenced religious behavior of adolescents, county level influences were weak [32]. In their study of parent and peer relationships and relational spirituality in adolescents and young adults, Desrosiers et al. [34] showed that parents and peers, particularly maternal communication and paternal affection, facilitated the development of relational spirituality.

With specific focus on the Chinese culture, Shek [18] reviewed ecological factors that influence the development of meaning in life among Chinese adolescents. Regarding the sociodemographic correlates of meaning in life in Chinese adolescents, it was found that gender, age, and economic disadvantage were related to adolescent life meaning, although the effect sizes were small. For example, regarding gender differences, there are research findings showing that male adolescents displayed a higher level of life purpose than did female adolescents, although such gender differences are not consistent across studies [18]. Within the family context, two types of family experiences that may shape the meaning of life in adolescents are dyadic family processes (e.g., parent-child relationship and marital quality of the parents) and systemic family attributes (e.g., family functioning and communication patterns). Shek [18] reported that there were several cross-sectional studies showing that the quality of parenting was positively related to adolescent meaning of life indexed by the Chinese Purpose in Life Questionnaire. Besides, in a series of studies examining the relationship between family processes and adolescent development, positive parenting attributes (such as parental support and involvement) were related to existential well-being in several samples. There are longitudinal research findings showing that parenting characteristics and parent-adolescent conflict were related to adolescent life meaning. Finally, research evidence also supporting that family functioning is related to adolescent meaning in life, both cross-sectional and over time.

## 6. Spirituality and Adolescent Developmental Outcomes

Regarding the relationship between spirituality and quality of life, there are four possibilities. First, spirituality is a cause of quality of life. Second, spirituality is a concomitant of quality of life. Third, spirituality is a consequence of quality of life. Finally, spirituality and quality of life are moderated and/or mediated by other factors. While studies have been conducted to examine the first two possibilities, research on the latter two possibilities is almost nonexistent [15, 18].

There are theoretical accounts suggesting that spirituality is an antecedent of quality of life (i.e., first possibility). In the theory of logotherapy proposed by Frankl [35], it is asserted that when there is existential vacuum (i.e., loss of meaning in life), mental problems come in to fill the vacuum. Frankl's conceptualization about human nature is based on the premise of “will to meaning”. When a person fails to find meaning in life and a state of vacuum of perceived meaning in personal existence (i.e., existential vacuum) is present, he or she is confronted by “existential frustration”, which is characterized by the feeling of boredom [36]. Although the occurrence of existential vacuum does not necessarily lead to noogenic neuroses, it was contended that existential vacuum is an etiological factor of psychopathology. Based on the above reasoning, it could be assumed that purpose in life is causally related to adolescent developmental outcomes. In a review of the relationships among meaning in life and well-being, psychopathology, and spirituality, research shows that people experiencing greater life meaning report greater well-being, less psychopathology, and more positive experience of spirituality [37]. Emmons also argued that religion provides goals and value system contributes to life meaning which would eventually shape different aspects of a person's life [38].

In the area of adolescent spirituality, despite their findings that spiritual attributes were related to global and life domains, Sawatzky et al. [39] commented that there are few studies on spirituality and quality of life in adolescents and the mechanisms underlying the relationship remain relatively unknown. They remarked that “few studies have examined the relevance of spirituality in adolescents with respect to their quality of life (QOL), despite empirical literature suggesting that religion and spirituality are important to adolescents” (p. 6).

Rew and Wong reviewed the association between religiosity/spirituality and adolescent health attitude and behavior [40]. The review showed that although roughly half of the studies indicated that religiosity/spirituality had positive effect on adolescent health attitude and behavior, there were theoretical and methodological limitations of the studies. In a review of research on adolescent religiosity and mental health, Wong et al. [41] found that most studies showed a positive relationship between religiosity/spirituality and adolescent mental health. Cotton et al. reviewed religiosity/spirituality and health outcomes [42]. They differentiated distal domains (service attendance, frequency of prayers and meditation, self-rated religiosity) and proximal domains (meaning and peace, religious coping, church support) and reviewed the related studies on adolescent developmental outcomes. While studies showed negative relationship between religiosity/spirituality and adolescent health risk, positive relationships between religiosity/spirituality and physical/mental health were reported.

Reviews showed that spiritual well-being is positively related to health outcomes, although there are possible confounding effects in the reported relationships [43]. Regarding the relationship between spirituality and physical health, Powell et al. tested nine hypotheses with reference to mediated models (evaluation of the impact of religion or spirituality on health, regardless of whether or not such a relationship was mediated by established risk/protective factors) and independent models (evaluated religion or spirituality as an independent protective factor after controlling other effects) and concluded that church/service attendance protects healthy people against death [44]. Meanwhile, the authors also pointed out the need for more methodologically sound studies in the field.

The role of spiritual intervention has also received increasing attention in the literature. On one hand, patients expect helping professionals to address their spiritual needs [43]. On the other hand, different professional bodies give more attention to spiritual care. For example, the National Consensus Project for Quality Palliative Care regarded spiritual, religious, and existential aspects of care as a domain of quality palliative care requiring spiritual care (Domain 5). In addition, the White House Office of Faith-Based and Community Initiatives was established in the Bush administration. Theoretically, Lent argued that it is important to understand spiritual variables such as meaning in life so that client growth and rehabilitation can be promoted [45].

Under the assumption that spirituality influences health outcomes, spiritual intervention with the aims of treatment or restoration and improvement of quality of life has been developed. In a meta-analysis of 51 samples from 46 studies examining psychotherapies in which religious or spiritual (R/S) beliefs are incorporated, Worthington et al. [12] drew several conclusions. First, compared with patients receiving secular psychotherapies, patients receiving R/S psychotherapies had better improvement in psychological and spiritual outcomes. Second, in contexts where spiritual outcomes are important, psychotherapies with R/S are a treatment of choice. Third, practitioners could consider offering psychotherapies with R/S to highly religious or spiritual patients. 

With specific reference to the Chinese culture, there are research findings showing that purpose in life was negatively associated with psychological symptoms, including general psychological problems, trait anxiety, depression, and hopelessness. Furthermore, participants with different existential statuses also displayed different levels of psychological symptoms. There are also longitudinal data showing the adverse relationship between purpose in life and psychological symptoms over time. Besides psychiatric symptoms, meaning in life was found to be related to positive mental health measures [15, 18]. Shek et al. [46] also reported that the spirituality subscale score of the Chinese Positive Youth Development Scale was positively associated with other positive youth development constructs, including bonding, resilience, social competence, emotional competence, cognitive competence, behavioral competence, moral competence, self-determination, self-efficacy, beliefs in the future, clear and positive identity, recognition for positive behavior, prosocial involvement, and prosocial norms. These findings are generally consistent with the views of Ryff and Singer [19] that sense of meaning and sense of self-realization are two key components of positive mental health, where meaning in life provides the necessary inner resources to fuel optimal functioning. There are also research findings showing that meaning in life was related to prosocial behavior and antisocial behavior while negatively associated with problem behavior.

Consistent with this notion, there are research findings suggesting that meaning in life is an important factor in helping adolescents to face adversity. Shek [47] showed that adolescents with stronger endorsement of positive Chinese beliefs (or weaker endorsement of negative Chinese beliefs) about adversity generally had better psychological well-being and school adjustment and less problem behavior. Although adolescents' degree of agreement with Chinese cultural beliefs about adversity was generally associated with adolescent adjustment, this relationship was stronger in adolescents with economic disadvantage than in adolescents without economic disadvantage. Nevertheless, while this study is pioneer in Hong Kong, replication of the findings is necessary in view of the worsening of income disparity and inequality in Hong Kong.

## 7. Promotion of Spirituality in Adolescents

Given the importance of spirituality, there are several ways to promote adolescent spirituality. The first strategy is to understand different forms of religions and spirituality via different media, including print and nonprint media. Enhanced understanding is important as far as religious and spiritual beliefs are concerned. However, understanding alone is not enough. Active reflection and experience are important processes in the development of spirituality. “Why do we exist? Where are we going? Is there any life after death? What should we do when we are still conscious?” These are important spiritual questions demanding conscious reflection. Besides gaining more experience and having personal reflections, joining religious groups, church activities, and spiritually related gatherings provide a good opportunity to develop spirituality. Bruce and Cockreham proposed different ways of promoting spirituality in adolescent girls via group work approach [48]. Besides, as significant-others surrounding adolescents (such as parents, teachers, and peers) have important influence on adolescent spirituality, how to shape adolescent spirituality through such significant personal relationships could be considered.

Finally, curricular-based programs can be utilized to promote spirituality in adolescents. For example, Hui and Ho [49] evaluated a forgiveness training program via quantitative and qualitative methods. Although there was no significant improvement in self-esteem and hope among the participants based on the pretest and posttest scores, participants showed better conception of forgiveness and had a positive attitude to using forgiveness. They concluded that it was “viable to promote forgiveness as a classroom guidance program” (p. 477). In the Project P.A.T.H.S. which attempts to promote holistic development in Chinese adolescents, units on spirituality are included in the Secondary 1 to Secondary 3 curricula [50–52]. Finally, in the course entitled “Tomorrow's Leaders” developed in The Hong Kong Polytechnic University, the following elements pertinent to the construct of spirituality are included: definition and basic concepts of spirituality, theories of spirituality, antecedents of spirituality, spirituality and adolescent developmental outcomes, spirituality and leadership, and ways to promote spiritual leadership.

## 8. Existing Research Gaps and Future Research Directions

King and Boyatzis [5] described that adolescence is “an age period of intense ideological hunger, a striving for meaning and purpose, and desire for relationships and connectedness” (p. 2). Given its importance, what are the research directions as far as the study of adolescent spirituality is concerned? Conceptually speaking, although literature shows that ecological factors at the individual, interpersonal, and family contexts are related to adolescent spirituality, there are several conceptual gaps. First, although there are views suggesting that spirituality influences adolescent developmental outcomes, how developmental outcomes may influence the development of purpose in life is far from clear (i.e., bidirectional relationships between purpose in life and developmental outcomes). Obviously, accumulation of research findings in this area would help to enrich Frankl's idea on the role of existential vacuum in human behavior.

Second, based on the ecological model, further studies should be conducted to examine how individual factors (e.g., religiosity and values), family factors (e.g., global parenting versus specific parenting practice, behavioral control, and psychological control), and social factors (e.g., endorsement of Chinese superstitious beliefs) are related to adolescent spirituality. This research direction is consistent with the argument of Fry [33] that “whether adolescents' life meaning and wisdom will grow and unfold from being relatively straightforward to being mature and complex will depend invariably on the presence or absence of a number of other intervening and moderating influences and contextual factors” (p. 93). It would be theoretically interesting to look at the relationships among life meaning, character building and religiousness, and subjective well-being.

Third, although the present paper highlights the importance of family processes in adolescents' purpose in life, further work is needed to examine how specific family processes and related experiences are related to purpose in life among adolescents. For example, it would be interesting to study how purpose in life of the parents is related to that of their adolescent children. It is important to examine the achievement of life meaning through love in close relationships.

Finally, although there are research findings in the area of human development examining the influence of spirituality on developmental outcomes in different stages of life span, Ellison and Lee [53] stated that spiritual struggles, including troubled relationships with God, negative interaction in religious settings, and chronic religious doubting, were related to psychological distress. The possible “dark side” of adolescent spirituality should be considered.
